# Moral judgment modulates neural responses to the perception of other’s pain: an ERP study

**DOI:** 10.1038/srep20851

**Published:** 2016-02-11

**Authors:** Fang Cui, Ning Ma, Yue-jia Luo

**Affiliations:** 1 Institute of Affective and Social Neuroscience, Shenzhen University, Shenzhen, China; 2 Research Center of Sport Psychology, Wuhan Sports University, Wuhan, China

## Abstract

Morality and empathy are both crucial in building human society. Yet the relationship between them has been merely explored. The present study revealed how the morality influenced empathy for pain by comparing the ERPs elicited by pictures showing the targets’ in pain primed by different moral information about the targets. We found that when the target was a moral one or a neutral one, the painful pictures elicited significantly larger amplitude in N2 than the non-painful pictures, but when the target was an immoral one, the difference between the amplitudes of N2 component elicited by painful and non-painful pictures became insignificant. We proposed that this effect was induced by the decreased affective arousal when observing an immoral person in pain. The reduced neural response towards the immoral one’s pain can keep us alert when we face the potentially dangerous people thereby increasing our chance of survival. SLORTEA results showed the source of this difference in N2 localized in the ventral medial prefrontal cortex (vmPFC) and the rostral anterior cingulate cortex (rACC) areas.

Morality is a code of values and customs that guide social conduct, which is innate to the human brain^1^. Morality is a fundamental component of human cultures and has been defined as prescriptive norms regarding how people should treat one another, including concepts such as justice, fairness, and rights^2^. It seems that human beings always perceive and evaluate others intuitively as morally laden^3^,^4^. Studies have shown that when people form the first impression of a person or a group, they are more interested in their traits concerning morality than traits concerning competence and sociality^5^. Neuroimaging studies found two regions were critical in moral judgment: the amygdala and the ventral medial prefrontal cortex (vmPFC). The former one is mainly engaged in guiding behaviors based on bottom-up, stimulus-based information which is emotionally salient and the role of the latter one is more integrative and modulated by present goals^6^,^7^.One study found that viewing scenes that evoked moral emotions (e.g. physical assaults, poor abandoned children) activated the vmPFC^8^. And study comparing patients with bilateral damage of the vmPFC with neurologically normal control group found that for personal moral dilemmas, the patients with vmPFC damage were more likely to endorse the proposed action^9^.

Empathy is defined as the ability to vicariously share the affective states of others, thereby facilitating our comprehension of the affections, motivations and actions of others^10^,^11^. Neuroimaging evidence suggests that there are two components of empathy which are subserved by distinct brain networks^12^. The affective component of empathy has been framed as reflecting rapid bottom–up activation of subcortical/cortical circuitries with neural underpinnings in the mirror neuron system (i.e. intra-parietal lobule, inferior frontal gyrus (IFG) and dorsal premotor cortex) and the limbic system (i.e. amygdala, ACC, anterior insula and ventral striatum^13^,^14^,^15^,^16^. The cognitive component of empathy, on the other hand, has been shown to be influenced by higher-level, top–down, signals originating in prefrontal cortical circuitries^12^,^14^. ERP studies found that both early (N1, N2) and later (P3) ERP components were revealed from the comparison of observing others receiving painful stimuli to non-painful stimuli^17^,^18^,^19^. N2 component has been consistently reported in observing other’s physical pain and has been suggested as a biomarker of the affective component of empathy for pain^20^. The gender difference in empathy for pain was found on N2 component, where the females who were more empathic show enhanced amplitudes on N2 than males^43^. The amplitude of N2 was also significantly correlated with subjective rating of affective empathy and scores in Empathic Concern Scale^21^,^22^.

There is a complex relation between morality and empathy based on the literature from developmental, behavioral psychology, and social neuroscience^23^. Empathy plays an important role in morality by two ways: first, empathy allows humans to understand how others are emotionally affected by given actions, which can directly inform people to make moral decisions in the next step; secondly, empathy can motivate people to behave in accordance with moral principles such as maximizing the well-being of others or not inflicting harm to others^23^,^24^,^25^. Meanwhile, it is reasonable to suppose that morality also has a strong effect on empathy. When facing immoral persons, we would try not to get involved with their emotions and feelings since the immoral persons are more likely to be dangerous. Keeping alert and emotionless to immoral ones would be better for our own safety. This assumption was supported by the evidence that reduced empathic responses were observed when viewing unfair person’s pain, compared to viewing the pain of a fair one^26^. However, how morality modulates empathy has not been explored from the temporal aspect yet, as far as we know.

To address this question, the present study used a word-priming paradigm, where the participants were primed with words describing different moral information about the characters preceding pictures depicting people’s hand/forearm/foot under painful or non-painful conditions. There were three kinds of characters: the ‘moral’ one (Blood donor), the ‘immoral’ one (Killer) and the ‘neutral’ one (Unidentified). To explore the influence of morality on empathy, we compared the differences between the ERP components of different conditions during the presentation of the pictures. We hypothesized that if there was a reduced empathic responses when observing immoral person’s pain then a reduced N2 would be observed in the presentation of painful pictures primed by immoral cue (i.e. Killer) comparing to the presentation of painful pictures primed by moral and neutral cues (i.e. Blood Donor and Unidentified).

## Methods

### Participants

Twenty-eight right-handed participants (14 male, 21.54 ± 2.25y (mean ± S.E.)) with no history of neurological disorders, brain injury or developmental disabilities participated in the experiment. All of them have normal or corrected to normal vision. All participants signed an informed consent form before the experiment. The experiment was conducted in accordance with the Declaration of Helsinki (BMJ 1991; 302: 1194) and was approved by the Medical Ethical Committee of the Medical School of Shenzhen University, China.

### Stimuli

**Figure 1:**
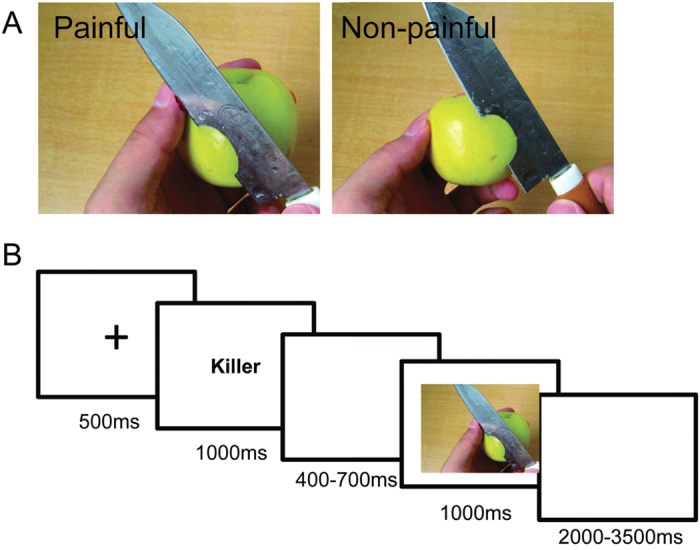
Experimental design (**A**) an example of the picture stimuli used in the experiment. The Left side shows a painful picture and the right side shows a non-painful picture. (**B**) an example of a single trial.

The stimuli used in the experiment were pictures showing a person’s hands/forearms/feet in painful or non-painful situations, which have been used in previous ERP studies. All the situations depicted in these pictures were ordinary events in daily life (Fig. 1A, Left). All the events showing in the non-painful pictures were corresponding to these in the painful ones just without the nociceptive component (Fig. 1A, Right). There were 60 painful pictures and 60 non-painful pictures in total. All of them had the same size of 9 × 6.76 cm (width × height) and 100 pixels per inch. 9-point Likert scales were used to evaluate the pain intensity, emotional valence and arousal level of each picture, the results of the evaluation can be accessed from the original papers^17^,^19^.

### Experimental procedures

Participants sat in a dark and quiet room alone during the recording. A 15-inch color monitor was placed in front of him/her. During the task, the pictures were presented in the center of a white background. Each stimulus was presented with a size of 22.5 × 16.9 cm (width × height), subtending a visual angle of 12.8° × 7.7°at a viewing distance of 100 cm.

It was a 3 × 2 within-subjects design. The first factor was moral information, which was manipulated by the priming words at three levels: the “Killer”, the “Blood Donor” and the “Unidentified”. The second factor was the pictures: “Painful” pictures and “Non-painful” pictures. During the recording the participants observed the pictures appearing randomly. Before the appearance of the pictures, participants would see a priming word informing them the moral information of the person in the following picture. The participants were instructed to image the person in the picture was the same character they were informed by the priming word. In order to help the participants imagine, before the recording, they were given two news reports to read: one describing the terrorists killing innocent passengers in a train station; the other one describing college students voluntarily donate their blood to help people.

In each trial, a fixation was presented for 500 ms, followed by the priming word lasting for 1000 ms. Then a blank interval was presented for 400 ms to 700 ms randomly. Subsequently, the picture was presented for 1000 ms. There was an interval of 2000 to 3500 ms between trials (Fig. 1B). In order to avoid the lack of attention due to the passive observation task, the participants were required to answer questions about the priming word or the picture randomly (12.5% of the trials were followed by a question). The participants were instructed to answer them by pressing the button “1” or “2” on a keyboard placed in front of them. For example, the question could be “What is the identity of the person?” Two choices appeared below the question: “Killer” and “Unidentified”. If they believed “Killer” was the correct answer, they would press button “1” otherwise they would press button “2”. There were 360 trials separated into 3 sessions evenly. Each single picture was repeated 3 times. There was a 30 seconds rest between sessions.

The participants were given a written instruction of the task and enough practice before the EEG recording to ensure the task was fully understood. The pictures appeared during the practice were excluded from the task. After the EEG sessions the participants were asked to subjectively evaluate the moral level of the three characters (Killer; Blood Donor; Unidentified) on a 9-point Likert scale (1: the most immoral; 9: the most moral; 5: neither immoral nor moral).

### EEG acquisition and analysis

Electroencephalography (EEG) data were recorded from a 64-electrodes scalp cap using the 10–20 system (Brain Products, Munich, Germany). Left side of mastoid was used as reference and the electrode on the medial-frontal site was used as ground. Three electrodes were used to measure the electrooculogram (EOG) (for horizontal EOG, electrode was placed on the outer canthus of the left eye; for vertical EOG, the electrode was placed below the left eye). EEG and EOG activity was amplified at 0.01 Hz ~ 100 Hz band-passes and sampled at 250 Hz. All electrode impedances were maintained below 5 kΩ.

EEG data were pre-processed and analyzed using Matlab R2011b (MathWorks, US) and EEGLAB toolbox^27^. EEG data at each electrode were re-referenced to the average of the left and right mastoids before further analysis. Then the signal passed with 0.01–30 Hz band-pass filter. Time windows of 200 ms before and 1000 after onset of picture stimuli were segmented from EEG. And the time window of 200 ms before and 1000 after the onset of the priming word stimuli were also segmented. EOG artifacts were corrected using an independent component analysis (ICA)^28^. Epochs with amplitude values exceeding ±50 μV at any electrode were excluded from the average. These epochs constituted 5 ± 2.7% of the total number of epochs.

Further statistical analysis was conducted in IBM SPSS Statistics 22 (IBM Corp., Armonk, NY, USA). For ERP data, based on previous ERP studies using the same stimuli and voltage scalp maps in the current results the following electrodes were included for further analysis: F4, FC4, C4,CP4, and P4 (five right sites); Fz, FCz, Cz, CPz, and Pz (five midline sites); F3, FC3, C3, CP3, and P3 (five left sites)^17^,^19^,^29^. Repeated measures ANOVA (2 (Picture: Painful/Non-Painful) ×3 (Moral information: Unidentified/Killer/Blood Donor) ×3 (regions: left/midline/right) ×5 (electrodes)) were performed for each component of the picture stimuli epochs and for each component of the priming word stimuli epochs separately. Mean amplitudes were obtained from each grand-averaged peak. Degrees of freedom for F-ratios were corrected according to the Greenhouse–Geisser method. Statistical differences were considered significant at *p* < 0.05; post-hoc comparisons were Bonferroni-corrected at *p* < 0.05.

Based on the scalp-recorded electric potential distribution, the standardized low resolution brain electromagnetic tomography (sLORETA) software was used to compute the cortical three-dimensional distribution of current density. The sLORETA method is a properly standardized discrete, three-dimensional (3D) distributed, linear, minimum norm inverse solution^30^,^31^. In order to visualize the likely neural generators of scalp voltages within these temporal windows where the significant effect were found, the grand average was imported into sLORETA and the source estimation during these time windows were generated.

## Results

### Behavioral data

In order to keep the participants attended, we randomly inserted questions in trials during the task. The percentage of trials with questions was 12.5%. The accuracy of all participants was 96.270 ± 2.81% (mean ± SE). The rating of moral information of “Blood Donor” was significantly higher than “Killer” (Blood Donor: 8.067 ± 0.214; Killer: 2.333 ± 0.237 (mean ± S.E.); *t*_(87)_ = 7.99, *p* < 0.001). We didn’t report the subjective rating of the “Unidentified” here because all of the first 9 participants raised the question about how to rate an unidentified character therefore we didn’t ask the latter 19 participants to rate it.

### ERPs

The grand averaged ERPs to pictures and priming words were computed separately for each condition. There are six conditions in total: “Unidentified” followed by painful picture (U_P); “Unidentified” followed by non-painful picture (U_NP); “Killer” followed by painful picture (K_P); “Killer” followed by non-painful picture (K_NP); “Blood Donor” followed by painful picture (B_P); “Blood Donor” followed by non-painful picture (B_NP).

#### ERPs to picture stimuli

ERPs for picture stimuli displayed a negative component from 120 ms to 170 ms (N1) over the frontal and central regions, a positive component from 180 ms to 230 ms (P2) over the central region, a negative deflection from 240 ms to 290 ms (N2) over the frontal and central regions, a positive component from 350 ms to 450 ms (P3) over the parietal area and a late positive deflection from 500 ms to 700 ms (LPC) over the central and parietal regions.

**Figure 2:**
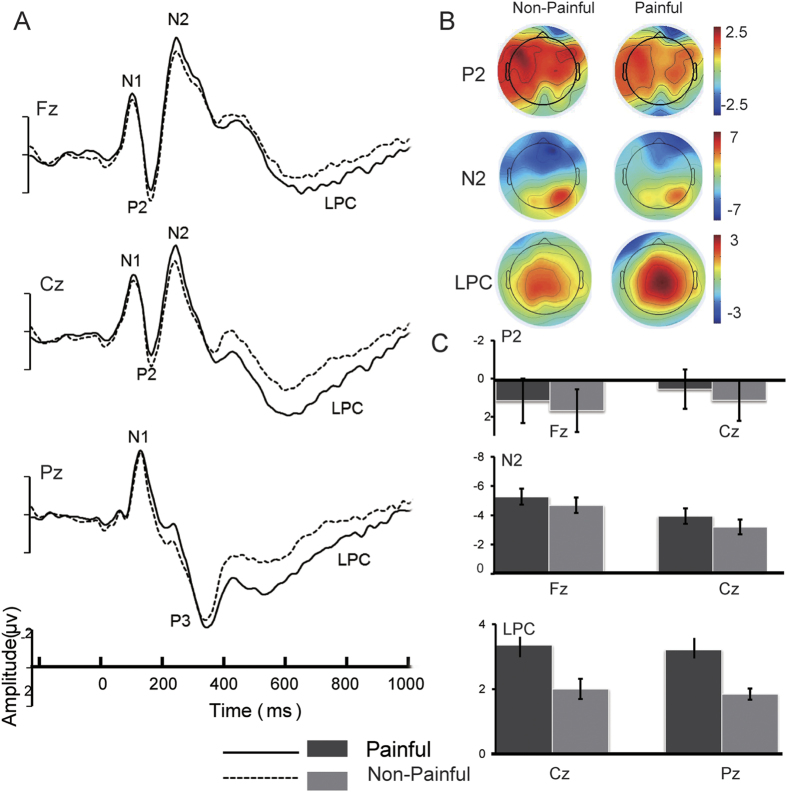
Cortical responses to painful and non-painful stimuli. (**A**) ERPs elicited by the painful stimuli were significantly decreased relative to those elicited by non-painful stimuli in the P2 window. ERPs elicited by the painful stimuli were significantly increased relative to those elicited by non-painful stimuli in N2 and LPC windows. (**B**) The Voltage scalp maps for P2, N2 and LPC in non-painful and painful conditions; (**C**) The averaged amplitudes within the P2, N2 and LPC time window in each conditions.

The main effect for pictures was significant in three components: P2, N2 and LPC. For P2 components, the non-painful pictures elicited a more positive deflection than painful pictures (*F*_(1, 27)_ = 10.273, *p* = 0.003). For region (*F*_(2, 54)_ = 4.279, *p* = 0.019) demonstrated P2 component, larger amplitudes were observed over the left than the right brain region (*p* = 0.020), but not the central region (*p* = 0.295). For N2 component, the painful pictures elicited a larger negative waveform than the non-painful pictures (*F*_(1, 27)_ = 19.263, *p* < 0.001). For region (*F*_(2, 54)_ = 8.077, *p* = 0.001) shown N2 component, the most negative deflection was found in the midline region than in the left and right region (*p* = 0.001, *p* = 0.012). For electrode (*F*_(4, 108)_ = 54.592, *p* < 0.001) in N2 component reveals the presence of N2 component over the frontal and central areas. For LPC component, the painful pictures elicited a larger positive deflection than the non-painful pictures (*F*_(1, 27)_ = 16.745, *p* < 0.001). For region (*F*_(2, 54)_ = 10.353, *p* < 0.001) shown LPC component, the largest positive deflection was found in the midline region than the left and right region (*p* < 0.001, *p* = 0.001). For electrode (*F*_(4,108)_ = 17.011, *p* < 0.001) reveals the presence of LPC in the central and parietal areas, but not in the frontal area (Fig. 2).

**Figure 3:**
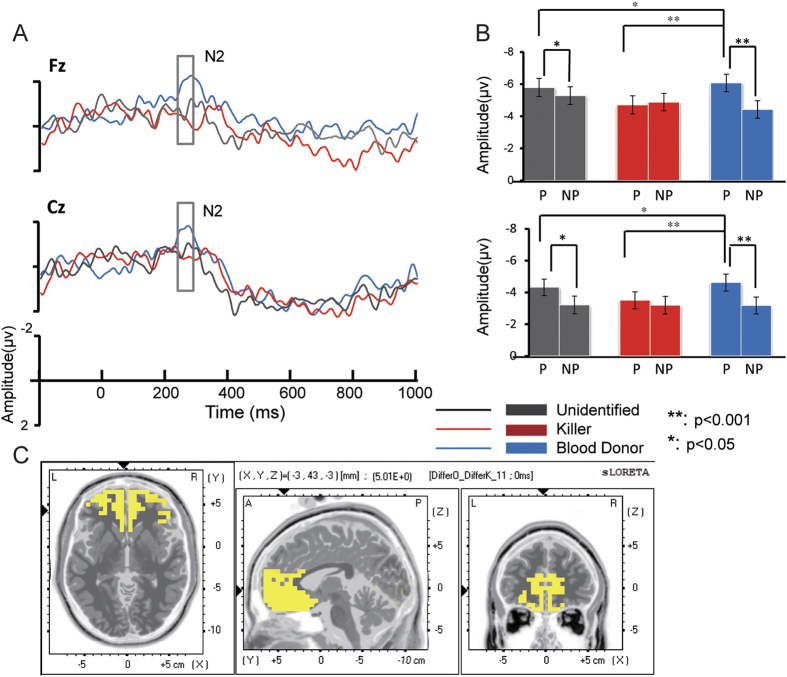
Difference waves to picture stimuli primed by different words (Grey: Unidentified; Red: Killer; Blue: Blood Donor). (**A**) The difference wave of (Painful – Non-painful) stimuli under three conditions at Fz and Cz sites. The Grey window illustrated the time window of N2. (**B**) The averaged amplitude under each condition (P: painful picture; NP: non-painful picture) at Fz and Cz sites. (**C**) sLORETA analyses revealed that the differences (Log of ratio of average),of dorsal ACC (BA32) and vmPFC (BA10) activation within the N2 window in response to painful picture and to non-painful picture was greater when the pictures were primed by “Blood Donor” than When the pictures were primed by “ Killer”.

Critically, the effect for morality on empathy was found in the N2 component. The Morality × Picture interaction (*F*_(2, 54)_ = 3.502, *p* = 0.044) indicated that the amplitude of N2 on pictures varied by moral information. By using pairwise comparisons, we found that when the priming word was “Unidentified” and “Blood Donor”, the painful pictures elicited a significantly more negative waveform than the non-painful pictures (*p* = 0.017, *p* < 0.001). However, the same comparison failed to reveal any significant differences between the two kinds of pictures on N2 when the priming word was ‘Killer’ (*p* = 0.244) (Fig. 3A).

In order to identify whether the change of difference between painful and non-painful pictures in different moral levels is due to the change in the painful stimuli or non-painful stimuli, or both, we separated the painful trials from the non-painful trials and ran two repeated measures ANOVAs (3 (Moral information: Unidentified/Killer/Blood Donor) ×3 (regions: left/midline/right) ×5 (electrodes)) for painful trials and non-painful trials separately on the N2 component. We found that there are no significant difference in non-painful trials on moral level but only in the painful trials, the main effect of moral information was significant (*F*_(2, 54)_ = 6.006, *p* = 0.005). The “Blood Donor” primed painful picture stimuli elicited a stronger negative amplitude than the “Unidentified” primed ones (*F*_(1, 27)_ = 12.475, *p* = 0.002) and the “Unidentified” primed ones elicited a stronger negative amplitude than the “Killer” primed ones (*F*_(1, 27)_ = 5.155, *p* = 0.031) (Fig. 3B).

It was worth noting that the stimuli associated by the priming label contain many attributes. The current design did not totally exclude the effects of the context rendered by the attributes other than morality (e.g., emotional valence and intensity). In order to clarify the influence of the priming’s valence and intensity has on the observed significant Morality × Picture interaction on N2, we re-contacted the 28 college students who had participated in the ERP study. We first refreshed them about the study then asked them to rate the intensity and valence of each priming word. To be more specifically, for the intensity, we asked them to rate from 1 to 7 (1: least intensive; 4: quite intensive, 7: very intensive); for the valence, they were also asked to rate from 1 to 7(1: very negative, 4: neutral, 7: very positive). We run repeated measures ANOVA with the subjective rating of intensity and valence of the priming words, separately. We found that the subjective rating of intensity and valence were significantly different among the three priming words (intensity: *F*(2, 27) = 1221.61, *p* < 0.001; Valence: *F*(2, 27) = 2262.26, *p* < 0.001). Pairwise comparison show that the rating of intensity of “Killer” and “Blood Donor” are significantly higher than the rating of intensity of “Unidentified” (Unidentified: 1.679 ± 0.146; Killer: 4.5 ± 0.227; Blood Donor: 4.892 ± 0.165 (mean ± s.e), *p* < 0.001, *p* < 0.001). The difference between the intensity of “Killer” and “Blood Donor” was not significant (*p* = 0.610). And the rating of valence of the “Killer” was significantly smaller (more negative) than the rating of “Blood Donor” and “Unidentified” (Unidentified: 3.75 ± 0.122; Killer: 2 ± 0.136; Blood Donor: 4.929 ± 0.162 (mean ± s.e), *p* < 0.001, *p* < 0.001). The rating of valence of “Unidentified” was significantly smaller than the rating of “Blood Donor” (*p* < 0.001).

Furthermore, in order to rule out the potential impact of the intensity and valence of the priming words on the amplitude of N2, we tried to run an ANCOVA to test whether the subjective rating of intensity and valence of the priming words were covariated with the amplitudes of N2. However, before the ANCOVA analysis, we found that the intensity and valence variables were not linearly related with amplitudes of N2 as the dependent variable, which did not meet the important assumption of ANCOVA. This indicated that the intensity and valence of the priming word did not impact on the observed N2.

#### Standardized low resolution tomography analysis (sLORETA)

Since the significant effect was found in the time window of N2 component (240 to 290 ms after the onset of stimuli), source estimation was conducted within this time window. We computed the cortical three-dimensional distribution of current densities of the two different waves between the Painful and Non-painful conditions primed by “Blood donor” and “Killer” (B_P>B_NP and K_P>K_NP), separately for all 28 subjects. Then we ran a paired t-test to compare the current density map generated by B_P>B_NP and K_P>K_NP and a number of brain areas in frontal lobe were revealed, including the vmPFC, orbitofrontal cortex (OFC), ACC and lateral PFC. The maximum sources were found in the ventral end of the pregenual cingulate cortex (pgACC, belong to the rostral ACC (rACC))^32^. (BA32, (MNI coordinates (x, y, z) = 3, 43, −3; Log of ratio of average = 1.051, p = 0.047) and vmPFC^33^ (BA10, MNI coordinates (x, y, z) = 9, 57, −8; Log of ratio of average = 1.047, p = 0.049) (Fig. 3C).

## Discussion

The present study utilized ERPs to investigate the modulation of morality on the perception of other’s pain. A word priming paradigm was applied by showing word to prime the moral information of the character in the following picture. There were three priming words, indicating three moral characters: the neutral one (Unidentified), the immoral one (Killer) and the moral one (Blood Donor) and two kinds of pictures: the painful ones and the non-painful ones. The significant effect of morality on empathy was found on the N2 component. We found an interaction effect of moral information × picture: when the priming word was the “Killer”, there was no significant difference between the painful and the non-painful pictures; when the priming words were “Unidentified” and “Blood Donor”, the amplitudes elicited by painful pictures on N2 was significantly larger than non-painful pictures.

Morality is a major component that forms the social norm and social expectancy^1^. Usually, moral judgments concern actions where one party harms or helps another, or treats a person or group fairly or unfairly^34^,^35^. What distinguish moral judgments from other items such as preference, aesthetics or non-moral good and bad is that moral judgments entail a belief that someone should be rewarded or punished^36^. Moral information may decrease the affective arousal and emotional contagion when observing the pain of the immoral targets. From an evolutionary point of view, if we reduce our emotional involvement with the immoral persons, we can be more alert when facing them. N2 component has consistently been found when participants observe other’s physical pain and it may index an early automatic component related to the sensitivity to other’s pain^20^,^37^. One ERP study found that the N2 component was enhanced when facing the pain of the participants’ own-race compared to other race. The reduced N2 possibly reflected suppressed affective responses towards other-races’ pain in the early stage and more sensitivity to own race member’s feelings^22^. In the current study, the source generators of the difference in N2 include the pgACC and vmPFC. This region was found to be consistently activated by negative affect and pain^32^. And the activity of pgACC has been reported to be significantly correlated with both the Balanced Emotional Empathy Scale (BEES)^38^ score and the Empathic Concern score of the Interpersonal Reactivity Index (IRI)^39^ in observing other’s pain^40^. It seems that the decreased activation of pgACC possibly indicated a reduced affective response to other’s pain. In addition, a series of neuroimaging evidence suggested that the vmPFC is involved in the emotional aspect of empathy^42^,^43^. Notice that in the current experimental settings we cannot ensure the participants actually “empathized” with the virtual characters. But when the participants were presented with emotionally salient pictures showing other’s pain, the affective arousal and the early emotional contagion process would be activated to facilitate the encoding of the stimuli. Therefore, we proposed that the reduced difference in N2 amplitude, as well as weaker activation of rACC and vmPFC reflect the decreased affective arousal and emotional contagion towards the immoral persons’ pain. However, due to the limitation of the spatial resolution of source localization in ERP studies. Future studies may need to replicate the present findings with fMRI and investigate the neural localization and functional connectivity of the effect of moral modulation on empathy.

In summary, our ERP findings highlight the neural mechanisms underlying the modulation effects of morality on the perception of other’s pain by finding that when the person in pain is immoral, the difference in brain responses to painful and non-painful stimuli would decrease compared to when the person is moral or neutral. This effect may be induced by less affective arousal and emotional contagion towards immoral person’s pain. The brain regions that are responsible for this effect would be the vmPFC and the pgACC. The differences in facing moral or immoral persons’ pain can keep us alert when facing people who are potentially dangerous and might harm us.

The present study has some important limitations. The results indicated a decreased affective arousal of other’s pain primed with the immoral information, however, the current study did not explore the neural correlates of the priming moral information, and this may raise concerns on whether the findings reflect modulation effects of morality only or combining with other effects, such as the lasting effect of the priming. Future study would be better to put effort on excluding the effects of the context and priming of moral information during empathy.

## Additional Information

**How to cite this article**: Cui, F. *et al.* Moral judgment modulates neural responses to the perception of other's pain: an ERP study. *Sci. Rep.* **6**, 20851; doi: 10.1038/srep20851 (2016).
